# Survival After Minimally Invasive vs. Open Radical Hysterectomy for Cervical Cancer: A Meta-Analysis

**DOI:** 10.3389/fonc.2020.01236

**Published:** 2020-07-24

**Authors:** Yizi Wang, Bo Li, Fang Ren, Zixuan Song, Ling Ouyang, Kuiran Liu

**Affiliations:** Department of Obstetrics and Gynecology, Shengjing Hospital of China Medical University, Shenyang, China

**Keywords:** cervical cancer, minimally invasive surgery, open surgery, radical hysterectomy, meta-analysis

## Abstract

**Background:** The comparison of survival outcomes between minimally invasive surgery and open surgery for cervical cancer patients remains controversial. We evaluated the survival outcomes of cervical cancer patients who underwent different surgical approaches.

**Methods:** A literature search was performed in PubMed, Embase, and Cochrane databases up to February 2020, using the MESH terms “minimally invasive surgical procedures” and “Uterine Cervical Neoplasms.” Included were all original comparative studies and trials both published and unpublished in English that were related to minimally invasive surgery and open surgery for cervical cancer patients with International Federation of Gynecology and Obstetrics (FIGO) 2009 stage < IIB. Begg's and Egger's regressions were used to evaluate publication bias.

**Results:** This meta-analysis included 28 studies enrolling 18,961 patients with cervical cancer. The overall analyses indicated that cervical cancer patients with FIGO 2009 stage < IIB who underwent minimally invasive surgery had a lower rate of OS (HR = 1.43, 95% CI = 1.06–1.92, *P* = 0.019) and DFS (HR = 1.50, 95% CI = 1.21–1.85, *P* < 0.001) than those who underwent open surgery. Moreover, minimally invasive surgery could lower OS (HR = 2.30, 95% CI = 1.50–3.52, *P* < 0.001) and DFS (HR = 1.94, 95% CI = 1.36–2.76, *P* < 0.001) of cervical cancer patients with FIGO 2009 stage ≤ IB1 compared to open surgery. However, there were no significant differences in OS (HR = 1.07, 95% CI = 0.65–1.76, *P* = 0.801) and DFS (HR = 1.20, 95% CI = 0.65–2.19, *P* = 0.559) in patients with tumors < 2 cm between the two groups.

**Conclusions:** Minimally invasive radical hysterectomy was associated with poor survival outcomes compared to open surgery. Patients with FIGO 2009 stage ≤ IB1 cervical cancer who underwent minimally invasive surgery have lower OS and DFS rates than those who underwent open surgery. Therefore, open surgery should be performed for cervical cancer patients. However, patients with tumors < 2 cm might take the most advantage of minimally invasive surgery without increasing poor prognosis. There are some limitations in the meta-analysis, which needs further high-quality multicenter studies to confirm and update our findings.

## Introduction

Cervical cancer is the fourth most common cancer and the fourth leading cause of cancer death in women worldwide (1). In 2020, it was estimated that there will be 13,800 new cases and 4,290 deaths in the United States, and in women aged 20–39 years, cervical cancer is the second leading cause of cancer death (2). Radical hysterectomy with pelvic lymphadenectomy is the standard recommended surgical treatment for early-stage cervical cancer patients. Traditionally, laparotomy has been deemed as the gold standard treatment for early cervical cancer (3). With the development of laparoscopic surgery, minimally invasive radical hysterectomy has ever been the standard surgical approach in patients with early-stage cervical cancer (4). Since 2018, the guidelines from the National Comprehensive Cancer Network (NCCN) advise that patients should be carefully informed about the risks and benefits of the different surgical approaches due to the findings of poorer survival outcomes with laparoscopy compared to laparotomy in the Laparoscopic Approach to Cervical Cancer (LACC) Trial (5). However, the latest guidelines from the NCCN advise that abdominal radical hysterectomy is the standard surgical treatment for early-stage cervical cancer patients (6).

Several meta-analyses have compared minimally invasive surgery (laparoscopic or robot-assisted radical hysterectomy) with open surgery (abdominal radical hysterectomy) in cervical cancer patients, showing that minimally invasive surgery is safe and has fewer perioperative complications and faster recovery than open surgery (7–9). Only a few studies included in previous meta-analyses looked at the rate of overall survival (OS) or disease-free survival (DFS), but neither laparoscopic nor robot-assisted radical hysterectomy has been associated with lower rates of OS or DFS (10–15). Instead, the evidence in support of minimally invasive surgery has been based mainly on observational studies.

A phase 3, multicenter, randomized trial of minimally invasive surgery vs. open surgery in patients with early-stage cervical cancer was published (5). The LACC trial showed that minimally invasive surgery could lower the rate of OS and DFS relative to open surgery in cervical cancer patients with International Federation of Gynecology and Obstetrics (FIGO) 2009 stage IA1 with lymphovascular space invasion (LVSI) to IB1. However, there were some limitations in the trial. The LACC trial didn't reach its preconcerted enrollment. And final results from LACC could not be generalized to patients with “low-risk” cervical cancer such as tumor size < 2 cm.

The oncologic outcomes of minimally invasive surgery compared to open surgery remain controversial. Therefore, we conducted a meta-analysis to observe OS and DFS in cervical cancer patients with FIGO 2009 stage < IIB between open and minimally invasive surgery, which might provide the evidence to choose the better surgical approach.

## Methods

### Search Strategy

This study was conducted in accordance with Preferred Reporting Items for Systematic Reviews and Meta-Analyses (PRISMA) guidelines, which was listed in Supplementary Table 1. We searched PubMed, Embase, and Cochrane databases for both published and unpublished trials up to February, 2020. The following MeSH and main keywords were used: “minimally invasive surgical procedures,” “minimally invasive surgery,” “procedure, minimal surgical,” “laparoscopy,” “robotic surgical procedures,” “robotic surgery,” and associated terms; and “uterine cervical neoplasms,” “cervical cancer,” “cancer of cervix,” “cervical neoplasm,” and associated terms. The language was restricted to English. For multiple-arm comparative studies, we extracted data only from the arms that matched our eligibility criteria. We also performed manual searches of the reference lists in the selected studies to retrieve all relevant data.

### Inclusion and Exclusion Criteria

Studies were selected according to PICOS (population, intervention, comparison, outcomes, and study design) guidelines if they met the following inclusion criteria: (1) population: cervical cancer patients with clinical FIGO 2009 stage < IIB; (2) intervention: radical hysterectomy was the primary treatment; (3) comparison: minimally invasive surgery vs. open surgery (both groups with or without adjuvant therapy); (4) outcomes: survival outcomes (OS and DFS) compared between two groups; (5) study design: studies were comparative (randomized control trials [RCTs] and observational studies).

Exclusion criteria were as follows: (1) population: patients with advanced cervical cancer who could not undergo surgery; (2) intervention: radiation or chemoradiation therapy was used as the primary treatment; (3) comparison: laparoscopic radical hysterectomy vs. robot-assisted radical hysterectomy or minimally invasive surgery vs. patients without open surgery; (4) outcomes: studies with insufficiently detailed data or lacking the outcomes of interest; (5) study design: single-arm study or review.

### Data Extraction and Quality Assessment of Included Studies

Two independent authors assessed the identified studies and the abstracts were reviewed to select full papers. All the authors evaluated the included studies for inclusion. The Jadad scale (16) and the Newcastle-Ottawa Scale (NOS) (17) were used to evaluate the quality of RCTs and observation studies, respectively. Discussion was performed among all the authors to resolve any disagreements.

### Statistical Analysis

The primary endpoints (time-to-event outcomes) of this meta-analysis were assessed using hazard ratios (HRs). If the HRs were not provided directly, we used Kaplan–Meier curves to get an estimated HR (18). Stata software, version 12.0 (2011; Stata Corp., College Station, TX, USA) was used to perform the meta-analysis. HRs are presented with 95% confidence intervals (CIs), and the two-tailed *P*-values of <0.05 were considered significant. We used Cochran's *Q*-test and the *I*^2^ statistic to evaluate the heterogeneity among the studies, and a *P* < 0.1 was considered as statistically significant (19, 20). The robustness of the results was assessed using sensitivity analyses (21). Finally, Begg's and Egger's regressions were used to evaluate publication bias (22, 23).

## Results

### Study Selection

**Figure 1:**
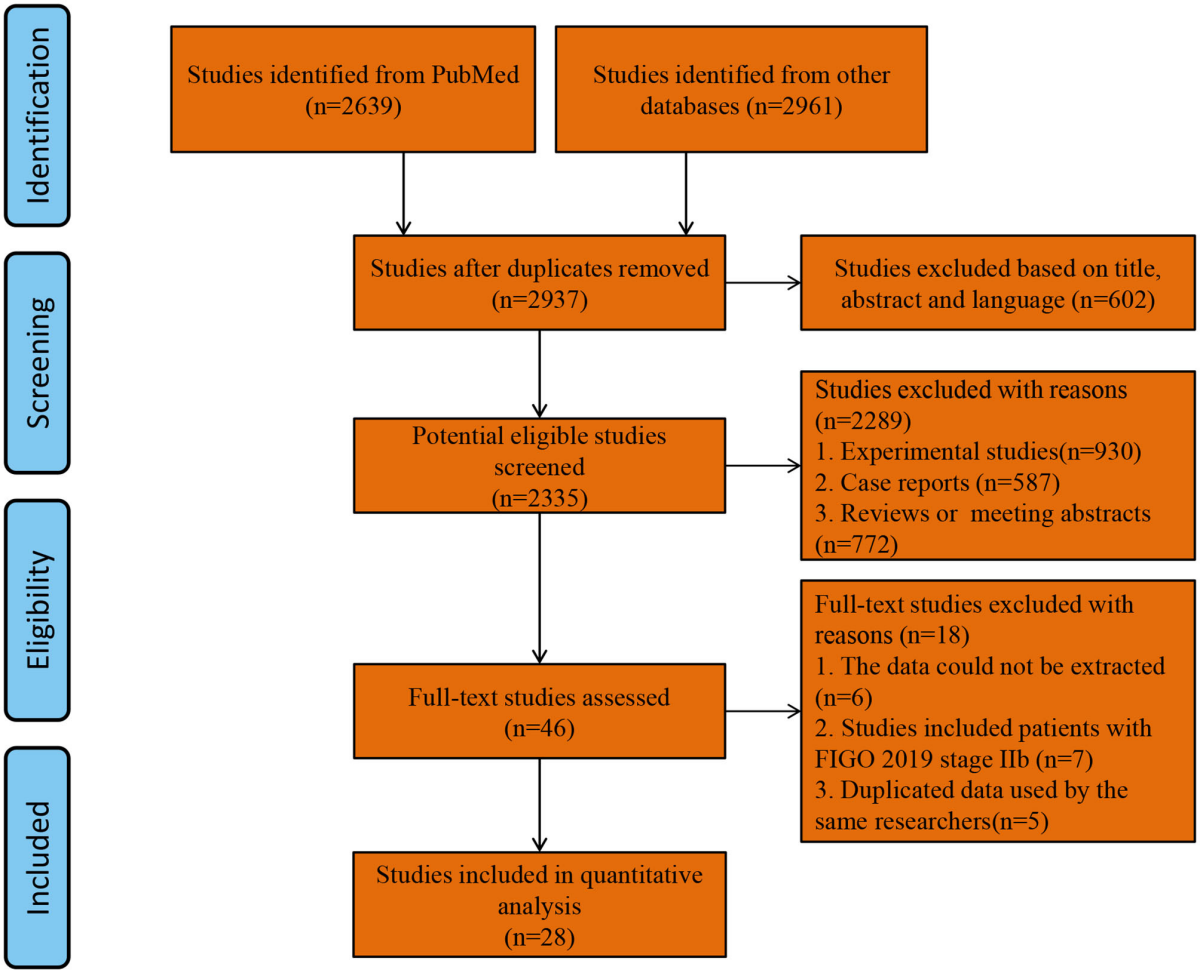
Flow diagram of the meta-analysis process.

Two thousand and nine hundred and thirty-seven studies were retrieved using our search strategy. After screening of the abstracts or titles, the full texts of 33 studies were further reviewed. Amongst these, five publications were excluded for the duplicated data used by the same researchers (24–28). Finally, 28 comparative studies which met the study inclusion criteria were selected for analysis (minimally invasive surgery group = 9,747, open surgery group = 9,214; total = 18,961 patients) (5, 10, 12–15, 29–50). A flow diagram of the meta-analysis process is illustrated in Figure 1. For one observational study in which the HR and 95% CIs were reported separately for laparoscopic surgery vs. open surgery and robot-assisted surgery vs. open surgery (37), we handled each surgical approach as a separate study in our meta-analysis. Tables 1, 2 show the main characteristics and quality scores of studies.

**Table 1:** Baseline characteristics of the included studies.

**Study**	**Country**	**Study type**	**Study**	**Setting**	**Study**
			**period**		**quality**
Uppal et al. (50)	USA	Observational	2010–2017	Multi	8
Pedone Anchora et al. (46)	Italian	Observational	NA	Multi	6
Service NCRaA (49)	England	Observational	2013–2016	Multi	8
Chiva et al. (48)	Europe	Observational	2013–2014	Multi	8
Paik et al. (30)	Korea	Observational	2000–2008	Multi	7
Yuan et al. (31)	China	Observational	2012–2014	Single	7
Cusimano et al. (32)	Canada	Observational	2006–2017	Multi	7
Kim et al. (33)	Korea	Observational	2000–2018	Single	7
Doo et al. (34)	England	Observational	2010–2016	Single	7
Lim et al. (35)	Singapore	Observational	2009–2014	Single	4
Alfonzo et al. (45)	Sweden	Observational	2011–2017	Multi	7
Ramirez et al. (5)	USA	RCT	2008–2017	Multi	7*
Melamed et al. (29)	USA	Observational	2010–2013	Multi	8
Guo et al. (36)	China	Observational	2008–2013	Single	4
Corrado et al. (37)	Italy	Observational	2001–2016	Multi	7
Kim et al. (47)	Korea	Observational	2011–2014	Multi	5
Wallin et al. (38)	Sweden	Observational	2006–2015	Single	6
Shah et al. (39)	USA	Observational	2001–2012	Multi	7
Wang et al. (40)	China	Observational	2002–2012	Single	6
Sert et al. (41)	USA	Observational	2005–2011	Multi	7
Zanagnolo et al. (42)	Spain	Observational	2006–2014	Multi	6
Ditto et al. (13)	Italy	Observational	2002–2013	Single	7
Kong et al. (14)	Korea	Observational	2006–2013	Single	6
Toptas et al. (43)	Turkey	Observational	2007–2010	Single	6
Nam et al. (12)	Korea	Observational	1997–2008	Multi	6
Lee et al. (10)	Korea	Observational	1994–2001	Single	6
Sobiczewski et al. (44)	Poland	Observational	2001–2004	Single	6
Malzoni et al. (15)	Italy	Observational	1995–2007	Single	6

*RCT, randomized controlled trial*.

* *Jadad scale was used to assess the quality of the randomized clinical trials*.

**Table 2:** Main characteristics of the study populations in the included studies.

**Study**	**Stage**	**Follow-up (median/mean months)**	**Total patients**	**No. patients in different groups**				**HR (95% CI)**	
				**Open surgery**	**Minimal invasive surgery**	**Laparoscopic surgery**	**Robotic surgery**	**OS**	**DFS**
Uppal et al. (50)	IA1–IB1	30.76	815	255	560	–	–	1.01 (0.5–2.2)	1.88 (1.04–3.25)
Pedone Anchora et al. (46)	IA1–IIA1	49	423	217	–	206	–	NA	0.86 (0.51–1.47)
Service NCRaA (49)	IA2, IB, IB1	37.2	929	365	564	–	–	4.0 (1.5–11.1)	NA
Chiva et al. (48)	IB1	58	245	122	123	–	–	4.25 (1.4–12.9)	1.94 (1.05–3.58)
Paik et al. (30)	IB–IIA	63.9	476	357	–	119	–	0.59 (0.07–4.92)	2.74 (1.33–5.65)
Yuan et al. (31)	IA2–IIA2	59	198	99	–	99	–	0.94 (0.42–2.09)	1.52 (0.80–2.89)
Cusimano et al. (32)	IB	72	958	483	475	–	–	2.20 (1.15–4.19)	1.97 (1.10–3.50)
Kim et al. (33)	IB1–IIA2	114.8	593	435	–	158	–	2.22 (1.12–4.41)	2.88 (1.71–4.86)
Doo et al. (34)	IB1	25.4	105	56	–	–	49	1.49 (0.26–8.65)	1.63 (0.68–3.90)
Lim et al. (35)	IA1–IIA	27	136	85	–	51	–	1.63 (0.48–5.49)	NA
Alfonzo et al. (45)	IA1–IB	44.5	864	236	–	–	628	1.00 (0.50–2.01)	1.08 (0.66–1.78)
Ramirez et al. (5)	IA1–IB1	30	631	312	319	244	45	6.00 (1.77–20.30)	3.74 (1.63–8.58)
Melamed et al. (29)	IA2,IB1	45	2,461	1,236	1,225	–	978	1.65 (1.22–2.22)	NA
Guo et al. (36)	IA1–IIA2	39	551	139	–	412	–	0.74 (0.37–1.45)	0.61 (0.29–1.30)
Corrado et al. (37)	IB1	41.7	341	101	–	152	88	2.56 (0.52–12.69)	0.47 (0.07–3.06)
Kim et al. (47)	NA	NA	6,335	3,235	–	3,100	–	0.74 (0.64–0.85)	NA
Wallin et al. (38)	IA1–IB1,IIA1	62.2	304	155	–	–	149	NA	2.13 (1.06–4.26)
Shah et al. (39)	IA1–IB2	NA	311	202	–	–	109	0.88 (0.23–3.32)	1.60 (0.75–3.43)
Wang et al. (40)	IA2–IIA2	68.33	406	203	–	203	–	0.77 (0.3–2.02)	0.98 (0.42–2.26)
Sert et al. (41)	IA1–IB2	39.6	491	232	–	–	259	2.0 (0.43–9.31)	1.3 (0.62–2.76)
Zanagnolo et al. (42)	IA2–IIA	41.64	307	104	–	–	203	1.33 (0.33–5.40)	0.84 (0.35–2.06)
Ditto et al. (13)	IA2,IB1	31	120	60	–	60	–	0.50 (0.07–3.77)	0.42 (0.10–2.00)
Kong et al. (14)	IB1,IIA	28	88	48	–	40	–	NA	0.28 (0–23.79)
Toptas et al. (43)	IA2,IB1	42.5	68	46	–	22	–	0.53 (0.01–22.5)	1.18 (0.28–4.96)
Nam et al. (12)	IA2–IIA	92	526	263	–	263	–	1.46 (0.62–3.43)	1.28 (0.62–2.64)
Lee et al. (10)	IA2–IIA	78	72	48	–	24	–	NA	0.72 (0.04–12.57)
Sobiczewski et al. (44)	IA,IB1,IIA	NA	80	58	–	22	–	NA	3.14 (0.67–14.73)
Malzoni et al. (15)	IA1–IB1	52.5	127	62	–	65	–	NA	1.15 (0.22–6.09)

*HR, hazard ratio; 95%CI, 95% confidence interval; OS, overall survival; DFS, disease-free survival; NA, not applicable*.

### Minimally Invasive Surgery vs. Open Surgery for Cervical Cancer

**Figure 2:**
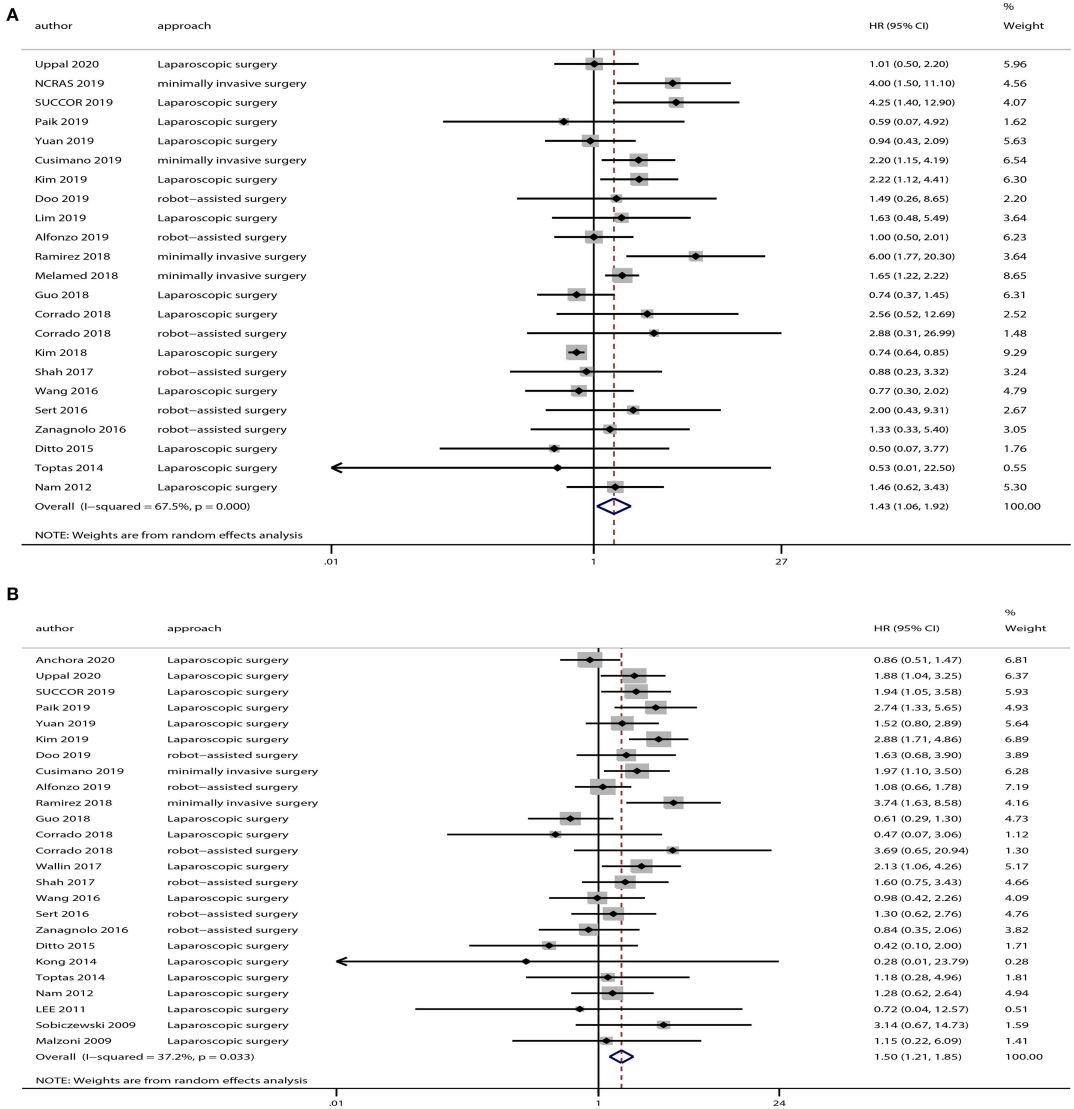
Overall analyses of minimally invasive surgery vs. open surgery for cervical cancer patients. **(A)** Overall survival; **(B)** disease-free survival.

The OS data was provided in 23 studies, and the HR was derived based on OS. Based on our pooled analysis, patients who underwent minimally invasive surgery had a lower rate of OS than those who underwent open surgery for cervical cancer (HR = 1.43, 95% CI = 1.06–1.92, *P* = 0.019; Figure 2A). In addition, 25 studies provided DFS data, and our pooled analysis indicated an inferior DFS in patients who underwent minimally invasive surgery than those who underwent open surgery (HR = 1.50, 95% CI = 1.21–1.85, *P* < 0.001; Figure 2B).

**Figure 3:**
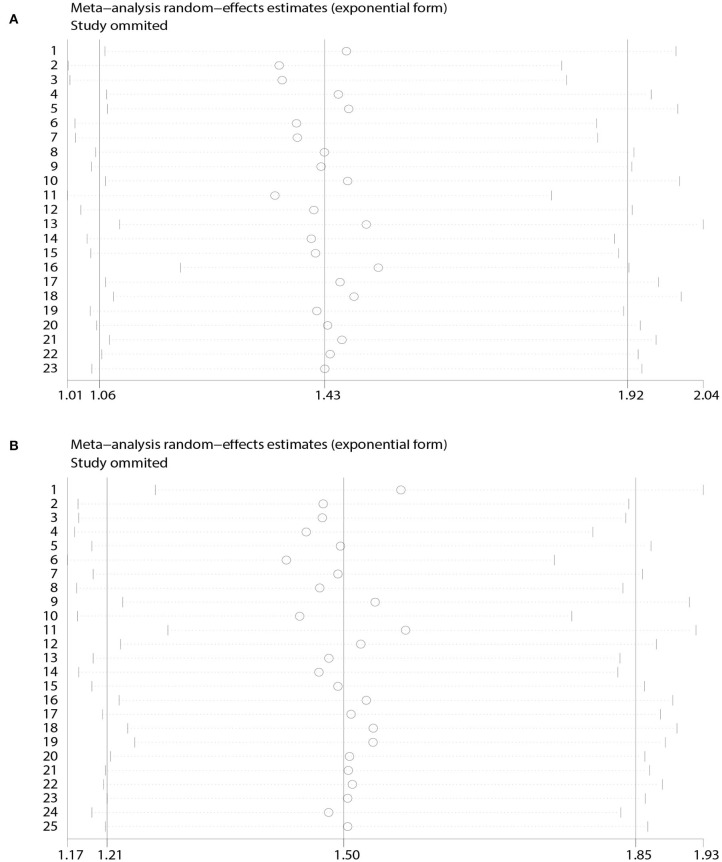
Sensitivity analyses based on **(A)** overall survival; **(B)** disease-free survival.

A heterogeneity was seen amongst the studies in terms of OS (χ^2^ = 67.64, *P* < 0.01, *I*^2^ = 67.5%) and DFS (χ^2^ = 38.24, *P* = 0.03, *I*^2^ = 37.2%). Hence, we conducted sensitivity analysis which showed that omitting any single study did not alter the corresponding pooled HRs of OS or DFS significantly (Figure 3).

**Figure 4:**
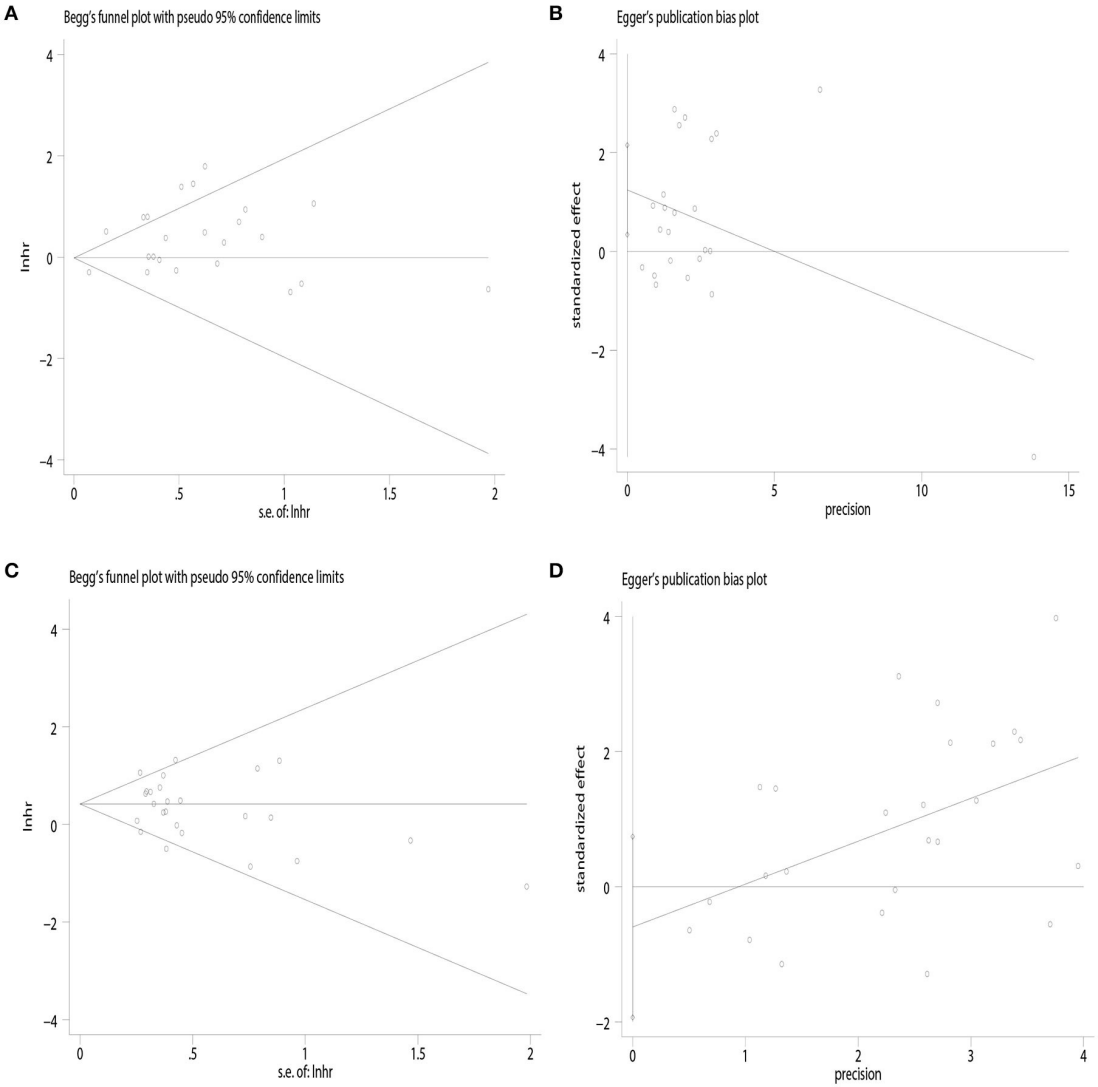
Publication bias. **(A)** Begg's test of overall survival; **(B)** Egger's test of overall survival; **(C)** Begg's test of disease-free survival; **(D)** Egger's test of disease-free survival.

The funnel plot showed potential publication bias in terms of OS [Begg's test: *P* = 0.67, (Figure 4A); Egger's test: *P* = 0.01, (Figure 4B)] but not of DFS [Begg's test: *P* = 0.41, (Figure 4C); Egger's test: *P* = 0.37, (Figure 4D)].

### Survival Outcomes for Patients With Stage ≤ IB1 Cervical Cancer

**Figure 5:**
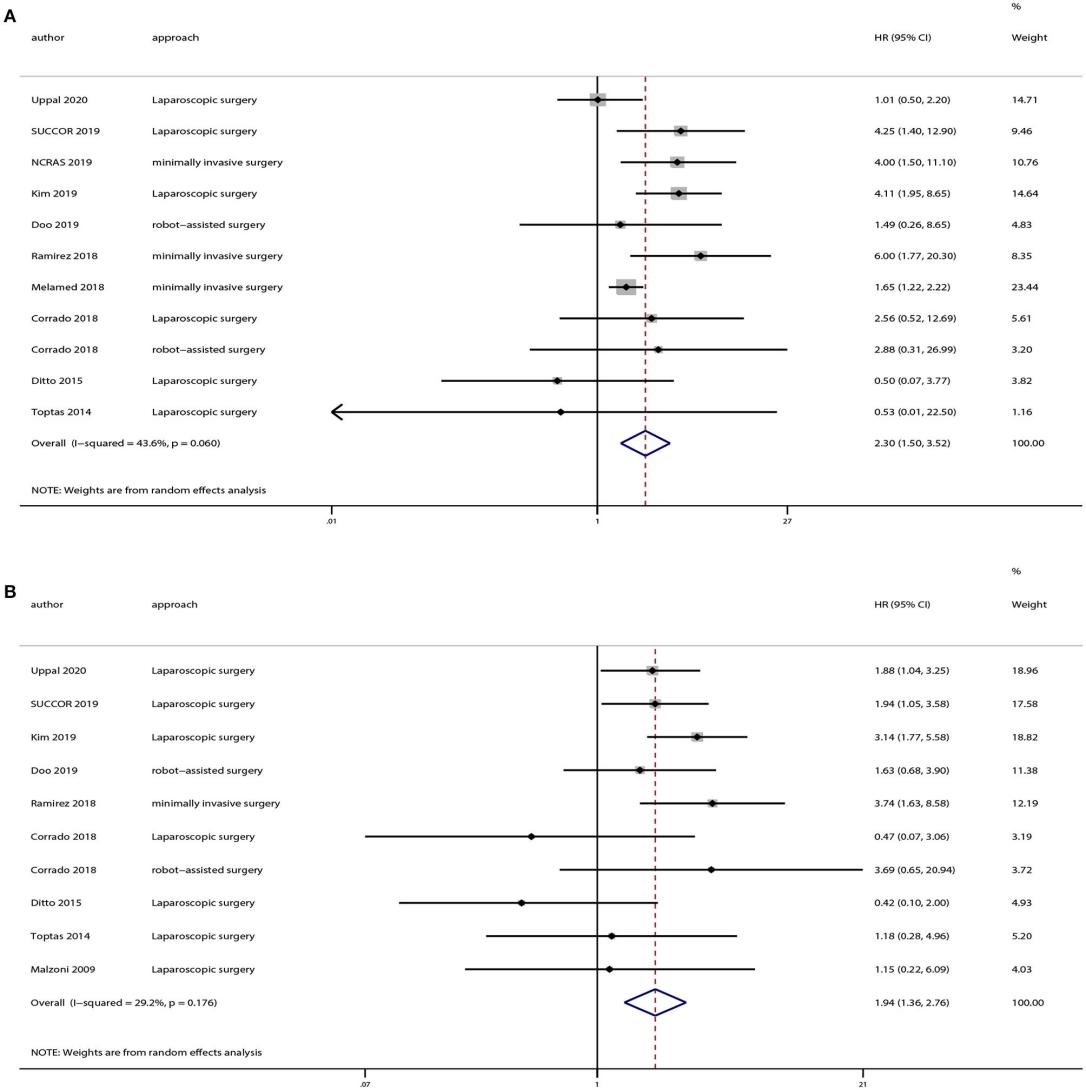
Subgroup analyses of patients with International Federation of Gynecology and Obstetrics (FIGO) 2009 stage ≤ IB1 cervical cancer between minimally invasive surgery group and open surgery group. **(A)** Overall survival; **(B)** disease-free survival.

We extracted OS and DFS data from the studies including patients with stage ≤ IB1 cervical cancer. And there were eight studies provided OS and DFS data of FIGO 2009 stage ≤ IB1. Our results demonstrated that patients in the minimally invasive surgery group had a lower rate of OS (HR = 2.30, 95% CI = 1.50–3.52, *P* < 0.001) and DFS (HR = 1.94, 95% CI = 1.36–2.76, *P* < 0.001) compared with those in the open surgery group, as shown in Figure 5.

### Subgroup Analyses Based on Tumor Dimension

**Figure 6:**
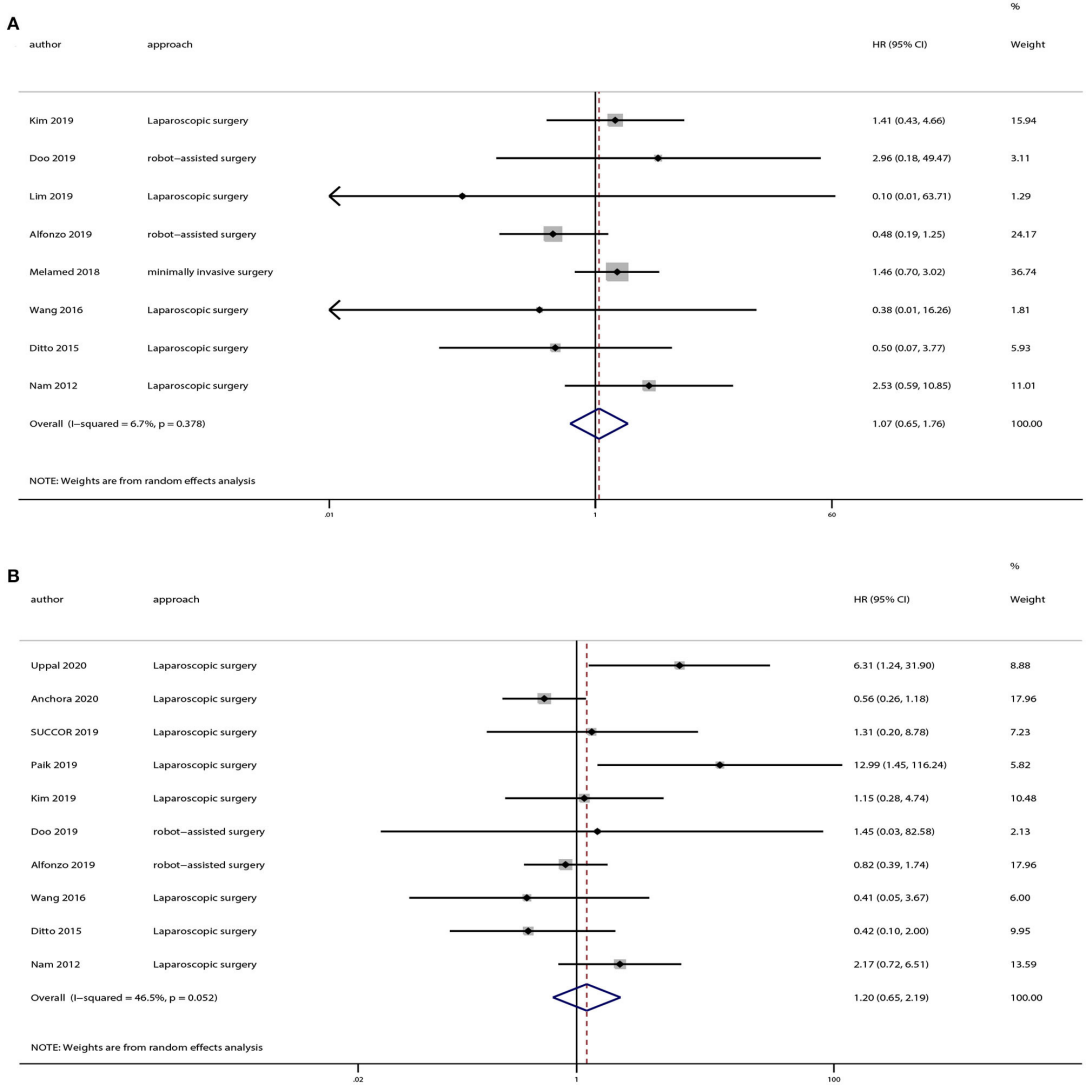
Subgroup analyses of patients with tumor size <2 cm between minimally invasive surgery group and open surgery group. **(A)** Overall survival; **(B)** disease-free survival.

**Figure 7:**
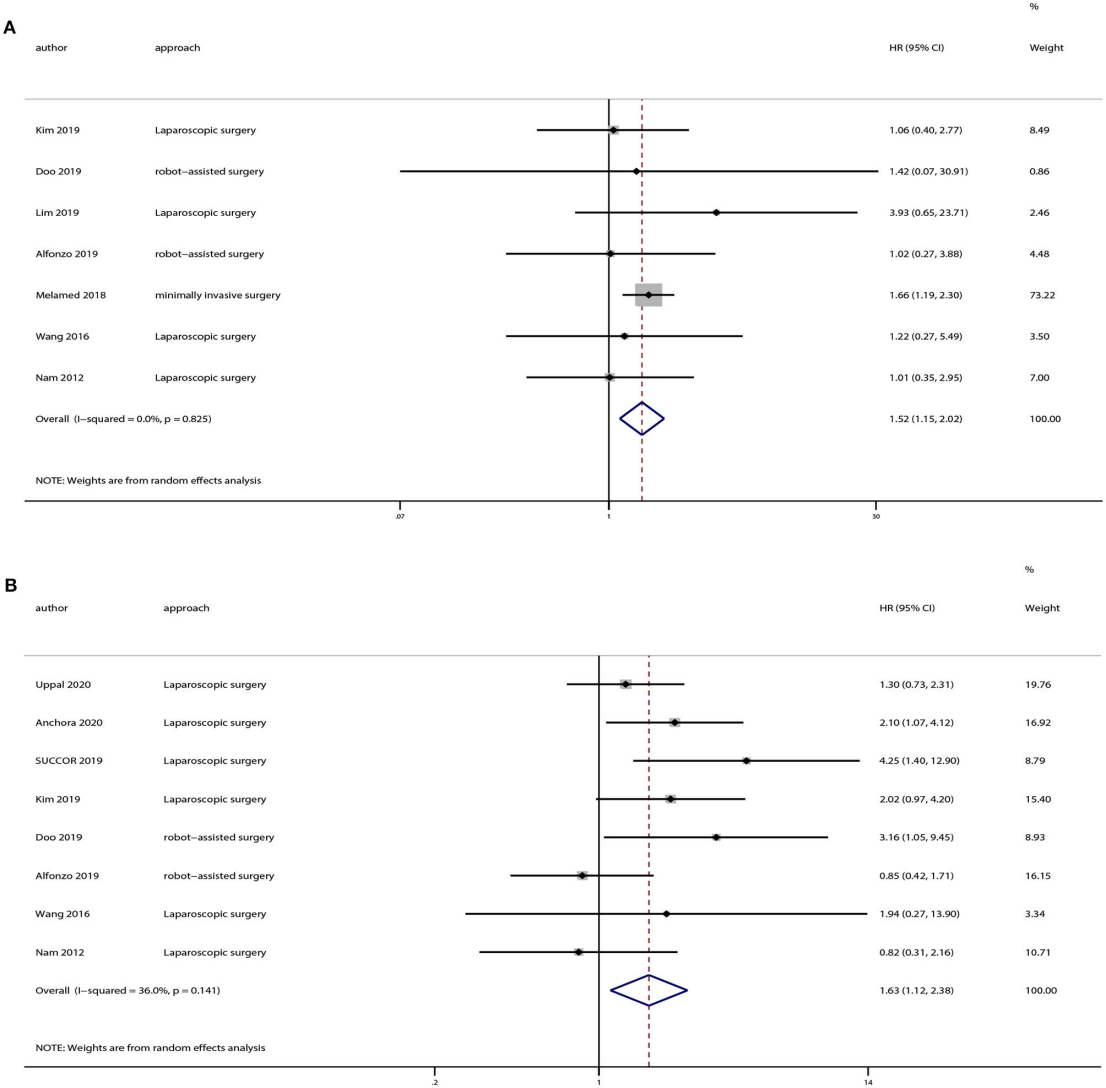
Subgroup analyses of patients with tumor size > 2 cm between minimally invasive surgery group and open surgery group. **(A)** Overall survival; **(B)** disease-free survival.

There were 12 strudies provided the data of tumors <2 or >2 cm. And we also extracted OS and DFS data from these studies. Eight studies provided OS and 10 studies provided DFS of tumors <2 cm, and the pooled results indicated no statistically significant difference in OS (HR = 1.07, 95% CI = 0.65–1.76, *P* = 0.801) and DFS (HR = 1.20, 95% CI = 0.65–2.19, *P* = 0.559) between the minimally invasive surgery group and open surgery group (Figure 6). With regard to patients with tumors >2 cm, seven studies provided OS and eight studies provided DFS. And the pooled results demonstrated that minimally invasive surgery could lower OS (HR = 1.52, 95% CI = 1.15–2.02, *P* = 0.003) and DFS (HR = 1.63, 95% CI = 1.12–2.38, *P* = 0.011) compared to the open surgery group (Figure 7).

## Discussion

Since 1975, survival rates have increased significantly in all of the most common cancers except for cervical and uterine cancer (51). Radical hysterectomy with pelvic lymphadenectomy remains the primary surgical treatment for cervical cancer (52). Since the first case of laparoscopic radical hysterectomy with pelvic lymphadenectomy was reported (53), minimally invasive surgery has developed gradually. Numerous studies (54–57) have stated its advantages of fewer perioperative complications and improved quality of life as compared with open surgery, but they did not report the oncologic outcomes.

Over the past decade, some studies have compared the minimally invasive and open approach, and found no differences in oncologic outcomes. In 2015, Wang et al. (8) and Cao et al. (9) performed separate meta-analyses evaluating the perioperative outcomes, efficiency, and prognostic results of traditional and minimally invasive techniques. However, these studies analyzed fewer than ten studies with survival outcomes, and all were based on retrospective cohorts. With the publication of the first RCT results of oncologic outcomes for different surgical approaches, the previous findings might be questioned. Thus, the time is right to evaluate systematically the survival outcomes associated with the minimally invasive approach.

Our meta-analysis included 28 studies enrolling 18,961 patients with cervical cancer. Based on our overall meta-analysis results, minimally invasive radical hysterectomy lowered the OS or DFS rate as compared with the open approach for patients with cervical cancer. Twenty-five studies reported DFS and 23 studies reported OS, including only one RCT. Ramirez et al. (5) reported that minimally invasive radical hysterectomy could lower the rate of OS and DFS as compared with the open approach. The RCT included women with stage ≤ IB1 cervical cancer and primarily evaluated survival outcomes. Hence, we analyzed the studies enrolling patients with stage ≤ IB1 cervical cancer, and found that the minimally invasive surgery group had a lower rate of OS and DFS in comparison with the open surgery group.

When compared to other prognostic stratification, the use of the tumor dimension appears to be the most reliable (46). And we also conducted subgroup based on tumor dimension. The results indicated an improved prognosis in patients with tumors >2 cm who underwent open surgery compared to those underwent minimally invasive surgery. However, there were no significant differences in OS or DFS in patients with tumors <2 cm between the two groups.

Before the LACC trial, a majority of the previous retrospective studies reached conclusions contrary to the RCT, we should consider the reasons why they may have done so. Open radical hysterectomy and pelvic lymphadenectomy treatment for cervical cancer has had a long history since the 1930s (58), and the minimally invasive approach was only reported much later, in the 1990s (53). During 2006–2010 (59), only 15.0% of all patients with cervical cancer who underwent radical hysterectomy underwent the minimally invasive approach, a proportion that increased to 45% during 2012–2015 (60). Most of the retrospective studies involved in our meta-analysis did not match the two groups in a same time frame, and open surgery was performed much more during an earlier time, when the criteria of adjuvant therapy was not defined clearly or carried out routinely (5). In addition, while small tumors would mostly likely be resected by the minimally invasive approach, more patients with large tumors may undergo open surgery (47). Differences in the tumor characters of the two surgery groups may have led to selection bias, resulting in a seemingly poorer survival outcome in the open surgery group. Meanwhile, we observed in many retrospective studies that patients who underwent minimally invasive surgery had a significantly shorter follow-up time than patients who underwent open surgery (11, 13, 14, 28, 37, 38, 41, 61). All of the above might create bias in calculating oncological outcomes.

On the other hand, when convinced by the result of the LACC trial by Ramirez et al. (5) or the recently high-quality observational studies (29), the latest NCCN guidelines have been updated to state that open abdominal surgery was the standard approach for radical hysterectomy. In terms of the poorer survival outcomes in the minimally invasive surgery group, we can offer some explanation. Some investigators have postulated that dissemination of malignant cells or increased lymph-vascular space invasion might occur with the use of the uterine manipulator (5, 62–64). And ESGO 2019 SUCCOR study showed significative difference in patients using or not a uterine manipulator (48). Meanwhile, experimental animal studies observed that CO_2_ pneumoperitoneum might promote intraperitoneal tumor dissemination or implantation (65–67). Finally, in the study by Sobiczewski et al. (44), we included, two patients in the laparoscopic surgery group were found to have intraperitoneal spread. However, with regard to the patients with tumor diameter smaller than 2 cm, we can't give a reasonable explanation for the non-significant difference between the two groups. And some authors explained that in case of larger tumors, the use of a uterine manipulator may squeeze them, which may result cancer spread (34, 46).

There are some limitations to our meta-analysis. First, only one RCT was included in the analysis. The majority of the studies involved were single center and retrospective observational studies with high risk for patients' selection bias, heterogeneity in the choice of postoperative therapy, and differences in surgeons' skills. Also, the criteria for candidate selection for radical hysterectomy may differ between centers and surgeons. The heterogeneity between-studies could have great influence in analyzing the median overall survival. Second, the reported tumor characteristics varied between studies, preventing independent comparisons of tumor size, histology, FIGO stage, and adjuvant treatment between the two groups. For example, some studies didn't state whether FIGO stage IA1 without LVSI is included (35, 36, 38, 39, 45). Most studies were not intended to analyze the impact of different type of radical hysterectomy on overall survival. And only a few studies stated that the patients were comparable in terms of histologic subtypes, rate of LVSI, tumor size, and grade and rate of use of adjuvant therapy (5, 13, 29, 34, 40, 45, 46, 50). Therefore, the results could not be combined because of such differences in the included studies. Third, when we analyzed the survival outcomes of patients with stage ≤ IB1 cervical cancer and tumor size by surgical approach, the number of studies included was relatively small. Fourth, although there was no significance in Begg's test based on the overall survival, Egger's test was statistically significant, which indicated a potential publication bias. Finally, data collected in our meta-analysis covered a particularly long timeframe during which minimally invasive surgery techniques have evolved considerably, which might not reflect changing survival outcomes over time.

## Conclusion

Minimally invasive radical hysterectomy was associated with inferior survival to open radical hysterectomy in patients with cervical cancer. At the same time, minimally invasive surgery may lower the rate of OS and DFS in comparison with open surgery for cervical cancer patients with FIGO 2009 stage ≤ IB1. However, patients with tumors <2 cm who underwent minimally invasive surgery didn't suffer inferior prognosis compared to those underwent open surgery.

## Data Availability Statement

All datasets analyzed for this study are included in the article/Supplementary Material.

## Author Contributions

YW and KL designed the research. YW and BL conducted the research and extracted the data. FR, ZS, LO, and KL evaluated the included studies and provided specific support in quantitative data analysis. YW drafted the first version of the manuscript. All authors read and approved the final version of the manuscript.

## Conflict of Interest

The authors declare that the research was conducted in the absence of any commercial or financial relationships that could be construed as a potential conflict of interest.
